# Genetic analysis of multiple synchronous lesions of the colon adenoma–carcinoma sequence

**DOI:** 10.1054/bjoc.1999.1091

**Published:** 2000-03-06

**Authors:** R Sedivy, B Wolf, M Kalipciyan, G G Steger, J Karner-Hanusch, R M Mader

**Affiliations:** Institute of Clinical Pathology, Department of Surgery, Department of Internal Medicine I, Division of Oncology, Vienna University School of Medicine, Waehringer Guertel 18–20, Vienna, A-1090, Austria

**Keywords:** adenoma–carcinoma sequence, aberrant crypt foci, carcinogenesis, colon cancer, mutation, microsatellite instability

## Abstract

The colorectal adenoma–carcinoma sequence represents a well-known paradigm for the sequential development of cancer driven by the accumulation of genomic defects. Although the colorectal adenoma–carcinoma sequence is well investigated, studies about tumours of different dignity co-existent in the same patient are seldom. In order to address the distribution of genetic alterations in different lesions of the same patient, we coincidently investigated carcinomas, adenomas and aberrant crypt foci in patients with sporadic colon cancer. By utilizing polymerase chain reaction, single-strand conformation polymorphism, heteroduplex-analysis, restriction fragment length polymorphism, protein truncation test and sequencing techniques we looked for mutations and microsatellite instability of APC, H- *ras*, K- *ras*, p53, DCC and the DNA repair genes *hMLH1/hMSH2*. In accordance with the suggested adenoma–carcinoma sequence of the colon, four patients reflected the progressive accumulation of genetic defects in synchronously appearing tumours during carcinogenesis. However, two patients with non-hereditary malignomas presented different genetic instabilities in different but synchronously appearing tumours suggesting non-clonal growth under almost identical conditions of the environment. Thus, sporadically manifesting multiple lesions of the colon were not necessarily driven by similar genetic mechanisms. Premalignant lesions may transform into malignant tumours starting from different types of genetic instability, which indicates independent and simultaneous tumorigenesis within the same organ. © 2000 Cancer Research Campaign


*British Journal of Cancer* (2000) **82**(7), 1276–1282
© 2000 Cancer Research Campaign
DOI: 10.1054/ bjoc.1999.1091, available online at http://www.idealibrary.com on IDEAL®

### Genetic analysis of multiple synchronous lesions of the colon adenoma–carcinoma sequence

**R Sedivy[1], B Wolf[2], M Kalipciyan[3], GG Steger[3], J Karner-Hanusch[3] and RM Mader[3]**

[1]Institute of Clinical Pathology, [2]Department of Surgery, [3]Department of Internal Medicine I, Division of Oncology, Vienna University School of Medicine, Waehringer Guertel 18–20, A-1090 Vienna, Austria

**Summary** The colorectal adenoma–carcinoma sequence represents a well-known paradigm for the sequential development of cancer driven by the accumulation of genomic defects. Although the colorectal adenoma–carcinoma sequence is well investigated, studies about tumours of different dignity co-existent in the same patient are seldom. In order to address the distribution of genetic alterations in different lesions of the same patient, we coincidently investigated carcinomas, adenomas and aberrant crypt foci in patients with sporadic colon cancer. By utilizing polymerase chain reaction, single-strand conformation polymorphism, heteroduplex-analysis, restriction fragment length polymorphism, protein truncation test and sequencing techniques we looked for mutations and microsatellite instability of APC, H-*ras*, K-*ras*, p53, DCC and the DNA repair genes *hMLH1/hMSH2*. In accordance with the suggested adenoma–carcinoma sequence of the colon, four patients reflected the progressive accumulation of genetic defects in synchronously appearing tumours during carcinogenesis. However, two patients with non-hereditary malignomas presented different genetic instabilities in different but synchronously appearing tumours suggesting non-clonal growth under almost identical conditions of the environment. Thus, sporadically manifesting multiple lesions of the colon were not necessarily driven by similar genetic mechanisms. Premalignant lesions may transform into malignant tumours starting from different types of genetic instability, which indicates independent and simultaneous tumorigenesis within the same organ. © 2000 Cancer Research Campaign

**Keywords:** adenoma–carcinoma sequence; aberrant crypt foci; carcinogenesis; colon cancer; mutation; microsatellite instability

The colorectal adenoma–carcinoma sequence describes the sequential development of cancer starting with aberrant crypt foci (ACF), which are supposed to be putative preneoplastic lesions (Pretlow et al, 1991). The consecutive step is thought to be the development of adenomas, which further increase in size and may finally develop to adenocarcinomas. This transition from benign precursors to malignant lesions is associated with the accumulation of genomic defects (Fearon et al, 1990). Thus, molecular analysis has identified inherited and somatic mutations that predispose to colon cancer (e.g. mismatch repair genes, *APC*, K-*ras* and *p53*). Defined molecular alterations have been linked to tumour progression in the cascade of histologically detectable lesions (for a recent review of the current models see Boland et al, 1998). Most recently, genetic abnormalities and microsatellite instability in colorectal cancer have been investigated in a large number of patients (Iniesta et al, 1998). There are, however, only sporadic data about genomic alterations in multiple lesions appearing synchronously in the same patient. Thus, we investigated multiple left-sided, synchronous lesions exploiting the fact that these lesions are embedded in the same environment and are exposed to almost identical influences. The investigated lesions included ACF, multiple adenomas of various sizes, adenocarcinomas, and one patient with additional liver metastasis. Using different molecular techniques, we evaluated microsatellite instabilities and mutations of APC, H-*ras*, K-*ras*, p53, DCC, and the DNA repair genes *hMLH1/hMSH2*.

*Received 10 February 1999*
*Revised 13 September 1999*
*Accepted 11 November 1999*

*Correspondence to:* RM Mader

### MATERIALS AND METHODS

To exclude any influence of tumor location as described for microsatellite instability, left-sided colon carcinomas were chosen for this study (Thibodeau et al, 1993). According to Bufill (1990), tumours located in the transverse colon nearby the splenic flexure were classified as being left-sided. ACF were located at least 10 cm away from the carcinoma.

### Patient characteristics

*Patient 1*

An 82-year-old woman with a 2 cm polypoid adenocarcinoma of the sigmoid (G1; pT1pN0pM0; Dukes' stage A) without adenoma, but several ACF.

*Patient 2*

A 47-year-old man with a 5 cm polypoid adenocarcinoma of the sigmoid (G1; pT3pN0pM0: Dukes' stage B). The tumour showed a mucinous component and presented lymphangiosis. In the rectum, a 1 cm tubulovillous adenoma with mild dysplasia was detected.

*Patient 3*

A 71-year-old woman with a 2 cm ulcerated adenocarcinoma of the rectum (G1; pT3pN0pM0; Dukes' stage B) presented a tubulovillous adenoma with moderate dysplasia (1 cm), and ACF located in the sigmoid.

*Patient 4*

A 27-year-old man with multiple colonic adenomas, and a 3 cm ulcerated adenocarcinoma of the rectum with regional lymph node

**Table 1** Characteristics of tumours

| Patient no. | Adenocarcinoma | | | | | Adenoma | | |
|---|---|---|---|---|---|---|---|---|
| | Anatomical segment | ∅ (cm) | Gross | Staging Grading | Dukes' stage | Anatomical segment | ∅ (cm) | Degree of dysplasia |
| 1 | Sigmoid | 2 | Polypoid | pT1N0M0 G1 | A | – | – | – |
| 2 | Sigmoid | 5 | Polypoid | pT3N0M0 G1 | B | Rectum | 1 | Mild |
| 3 | Rectum | 2 | Ulcerated | pT3N0M0 G1 | B | Sigmoid | 1 | Moderate |
| 4 | Rectum | 3 | Ulcerated | pT2N1M0 G1 | C | Descending<br>Descending<br>Sigmoid<br>Transverse | 3<br>2<br>1<br>0.5 | No. 1: moderate<br>No. 2.: moderate<br>No. 3.: mild<br>No. 4.: mild |
| 5 | Descending | 3 | Ulcerated | pT3N1M0 G1 | C | Descending | 1<br>0.5 | No. 1.: mild<br>No. 2.: mild |
| 6 | Transverse | 5 | Ulcerated | pT4N2M1 G1 | D | Descending | 1.5 | Severe |

ND = not detected, ∅ = diameter in centimetres, – = no material available.

metastases (G1; pT2pN1pMo; Dukes' stage C). In addition, less than 100 microadenomas and four larger tubulovillous adenomas with following characteristics were detected: adenoma 1 (3 cm in diameter) and adenoma 2 (2 cm in diameter) in the colon descendens, both with moderate dysplasia; adenoma 3 (1 cm in diameter) in the sigmoid, and adenoma 4 (0.5 cm in diameter) in the distal colon transversum (near splenic flexure), both with mild dysplasia.

### *Patient 5*

A 56-year-old man with 3 cm ulcerated adenocarcinoma of the colon descendens (G1; pT3pN1pMo; Dukes' stage C) with mucinous component. The regional lymph nodes were metastasized. Co-existent with the carcinoma, several ACF and two tubulovillous adenomas located in the colon descendens (1 cm and 0.5 cm diameter, both with mild dysplasia) were found.

### *Patient 6:*

A 66 year old man with a 5 cm ulcerated adenocarcinoma located in the distal third of the colon transversum near the splenic flexure (G1; pT4pN2pM1; Dukes' stage D), several ACF, and a tubulovillous adenoma with severe dysplasia in the colon descendens (1.5 cm). In addition to metastases of the regional lymph nodes, liver metastases were also present.

### Handling of tissue specimens

A total of 30 native tissue samples was obtained immediately after removal from six patients with colorectal neoplasms who underwent surgical routine intervention. Tissue strips of grossly normal mucosa of the resection margin were separated from uninvolved submucosa and muscularis propria. Tumour tissue was prepared from tubulovillous adenomas, colonic adenocarcinomas and, in one patient, from liver metastases of the corresponding colonic cancer. ACF were identified in whole mount preparations by staining the grossly normal colonic mucosa with methylene blue staining as previously described (Pretlow et al, 1991). ACF were distinguished from normal crypts by their deeper colour, larger size and round-oval shape of the luminal opening. Microdissection of ACF was performed on methylene blue-stained mucosa using a photomacroscope (Wild Heerbrugg, model M 400; Switzerland).

Histologically, ACF did not present dysplasia. Regional tumour-free lymph nodes were dissected from pericolonic tissue and were cleared from fatty tissue. For histopathologic examination, specimens were fixed in formalin. Five-micrometres serial sections of the paraffin-embedded material were stained with haematoxylin and eosin. All carcinomas examined were well differentiated adenocarcinomas of the colon. The tubulovillous adenomas presented a degree of dysplasia from mild to severe (Table 1).

As reference samples for tumour specimens, histopathologically tumour-free colonic mucosa, muscularis propria and lymph nodes of the same patient were used. To avoid cross contamination, each tissue sample was resected with freshly sterilized instruments.

Specimens were immersed into cell culture medium without fetal calf serum (RPMI-1640) and processed immediately. The specimens were divided into aliquots, shock frozen in a mixture of ethanol/dry ice, and were stored at –80°C until nucleic acid isolation. In addition, corresponding parts of all tissue samples intended for molecular analysis were paraffin-embedded for histopathologic examination.

### Isolation of genomic DNA

Frozen tissue was pulverized in a mortar cooled with liquid nitrogen. From this fine powder, genomic DNA was isolated using Genomix (Talent, Trieste, Italy). After photometric evaluation, genomic DNA was diluted to a final concentration of 0.2 $\mu g\ \mu l^{-1}$ in diethylpyrocarbonate-treated water and stored at –20°C.

### Analysis of dinucleotide repeat polymorphisms

Polymorphisms of CA repeat motifs were evaluated on chromosomes 2, 5, 17 and 18. On chromosome 2, CA repeat motifs on loci D2S123, D2S134 and D2S177 (Weissenbach, 1992*a*, 1992*b*, 1992*c*) proximal to the human DNA repair gene *hMSH2* were amplified by polymerase chain reaction (PCR) using the following primers: D2S123, + strand: AAA CAG GAT GCC TGC CTT TA, – strand: GGA CTT TCC ACC TAT GGG AC; D2S134, + strand: AAC GTC TGC TCG TCA GAG TC; – strand: CGA CTA CGT

© 2000 Cancer Research Campaign 

GCT GGC TAC TT; D2S177, + strand: AGC TCA GAG ACA CCT CTC CA; – strand: CTG TAT TAG GAT ACT TGG CTA TTG A. PCR was done as described below for the amplification of p53 using *Taq*-polymerase (Perkin-Elmer, Foster City, CA, USA) instead of Ampli Taq Gold-polymerase. With the exception of the primer annealing temperature, the cycling conditions were identical to those of p53 (D2S123: 54°C; D2S134: 58°C; D2S177: 55°C).

On chromosome 5, dinucleotide repeat motifs on loci D5S82 (Breukel et al, 1991*a*), D5S122 (Breukel et al, 1991*b*) and D5S346 (Spirio et al, 1991) proximal to the APC gene were amplified by PCR according to the literature.

On chromosome 17, a CA repeat motif (Jones and Nakamura, 1992) and a pentanucleotide repeat motif (Cawkwell et al, 1994) proximal to the *p53* gene were analysed.

On chromosome 18, a CA repeat motif on loci D18S34 (Weber and May, 1990) proximal to the *DCC* gene and an intragenic sequence containing a variable number of tandem repeats termed DCC-VNTR (Maesawa et al, 1995) were analysed. PCR products were then analysed on 7% polyacrylamide gels in 1 × TBE buffer and stained with ethidium bromide. Polymorphisms and deletions were determined by comparison of tumour samples with normal colonic tissue of the same patient.

### Single-strand conformation polymorphism of DCC and p53

Codons 138–473 of the *DCC* gene were amplified using four different pairs of primers and analysed according to the method of Miyake and co-workers (Miyake et al, 1994).

Similarly, exons 5–8 of p53 were amplified using the following intron-specific primers: exon 5, + strand: CTT TCA ACT CTG TCT CCT TCC TCT TCC TAC; exon 5, – strand: CTA AGA GCA ATC AGT GAG GAA TCA GAG G (PCR product: 297 bp); exon 6, + strand: CTG ATT CCT CAC TGA TTG CTC TTA GGT C; exon 6, – strand: CTG TGC AAT AGT TAA ACC CAT TTA CTT TGC (PCR product: 293 bp); exon 7, + strand: TGT TAT CTC CTA GGT TGG CTC TGA CTG TAC; exon 7, – strand: ATG AGA GGT GGA TGG GTA GTA GTA TGG AAG (PCR product: 242 bp); exon 8, + strand: TGG TAA TCT ACT GGG ACG GAA CAG C; exon 8, – strand: CTT CTT GTC CTG CTT GCT TAC CTC G (PCR product; 158 bp).

Amplification of the target sequences was carried out in a total volume of 50 µl containing the following reagents: 10 mM Tris–HCl (pH 8.3), 50 mM potassium chloride (KCl), 1.5 mM magnesium chloride ($MgCl_2$), 0.001% (w/v) gelatine, 0.25 µM of the forward and reverse primer, respectively, 0.5 µg genomic DNA, 0.2 mM dNTPs, 1.5 U Ampli *Taq* Gold-polymerase (Perkin-Elmer), overlaid with 50 µl mineral oil. In addition, the detergent W1 (0.05%; Gibco-BRL, Gaithersburg, MD, USA) was used when amplifying the exons of p53. Polymerase was activated by heat (10 min at 95°C). Then, 35–40 cycles were accomplished with a denaturation temperature of 94°C (1 min), annealing at 65°C (1 min) and extension at 72°C (30 s). After completion of the PCR cycles, the last extension time was 10 min at 72°C.

Successful amplification of DCC and p53 was confirmed by gel electrophoresis (1% agarose, Seakem GTG, Rockland, ME, USA) in 1 × TBE buffer, pH 8.0. For single-strand conformation polymorphism (SSCP) of both genes *p53* and *DCC*, amplification products were denatured in a formamide buffer (10 min at 95°C, rapidly cooled on ice), analysed on 10% polyacrylamide gels in 1 × TBE buffer, and evaluated after silver staining.

### Heteroduplex analysis of *APC*, *DCC* and *p53*

Heteroduplex analysis of the *APC* gene (fragments E, F and G of exon 15) was done as previously described (Friedl et al, 1993). For heteroduplex analysis of DCC and p53, one additional denaturing step was performed after completion of the PCR cycles (3 min at 99°C). Formation of heteroduplices was favoured by controlled cooling from 99°C to 10°C below the corresponding annealing temperature (0.6°C/min). Heteroduplices of APC, DCC and p53 were separated on 10% polyacrylamide gels in 1 × TBE buffer, and visualized by staining with Sybr Green I (Molecular Probes, Eugene, OR, USA) and epi-illumination at 254 nm wavelength.

### Sequencing of DCC

Following the Ampli cycle sequencing kit protocol (Perkin-Elmer), direct dideoxy-sequencing of the PCR products was performed on an automated DNA-Sequencer (Alf, Pharmacia, Uppsala, Sweden) using the 5′ terminally fluorescein isothiocyanate (FITC)-labelled sense or antisense strand PCR primers. Prior to sequencing, excess PCR-primers were digested using a mixture of 5 U exonuclease 1 and 5 U alkaline phosphatase (Amersham Life Sciences, Little Chalfont, UK).

### Restriction fragment length polymorphism of K-*ras* and H-*ras*

Mutations of K-*ras* (codons 12 and 13 of the first exon) and H-*ras* (codon 12 of the first exon) were evaluated by restriction fragment length polymorphism (RFLP) as previously described (Jiang et al, 1989). Briefly, exon 1 of K-*ras* and H-*ras* was amplified from gDNA of tumour samples and normal colonic mucosa by PCR. Mutations of K-*ras* were detected by agarose gel electrophoresis after digestion of the PCR products with *Bst*NI (codon 12) or *HphI* (codon 13) respectively. Similarly, mutations of H-*ras* were detected using *MspI* (codon 12).

### Protein truncation test for *hMLH1* and *hMSH2*

Using an in vitro transcription/translation assay, protein truncating mutations of the mismatch repair genes *hMLH1* and *hMSH2* were detected as previously described (Luce et al, 1995). Briefly, the coding sequence of the target was amplified by reverse transcription PCR (RT-PCR) using a + strand primer with a T7 promoter sequence and a translation initiation site, from which the protein was synthesized (reticulocyte lysate system, Promega, Madison, WI, USA). The tritiated protein ([$^3$H]-leucine) was analysed on a 12.5% SDS-polyacrylamide gel using Amplify (Amersham) to enhance the signal intensity.

### RESULTS

Investigating nine microsatellite markers, none of the malignant lesions showed microsatellite instability. It was thus concluded that all tumours were replication error phenotype negative (RER–).

 *© 2000 Cancer Research Campaign*

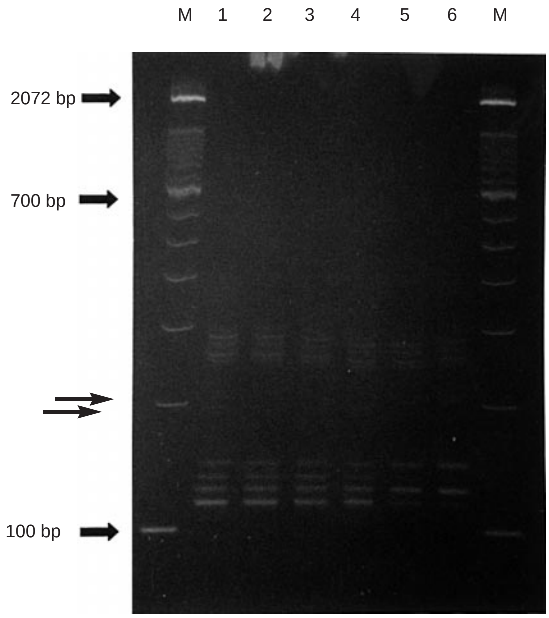

**Figure 1** Chromosome 5: dinucleotide repeats at the APC locus (D5S346, patient 4). Legend: 100 bp ladder (M), normal mucosa (lane 1), adenoma no. 1 (lane 2), adenoma no. 2 (lane 3), adenoma no. 3 (lane 4), adenoma no. 4 (lane 5), carcinoma (lane 6). Deletion of one allele is observed in adenoma no. 4 (lane 5) and the carcinoma (lane 6)

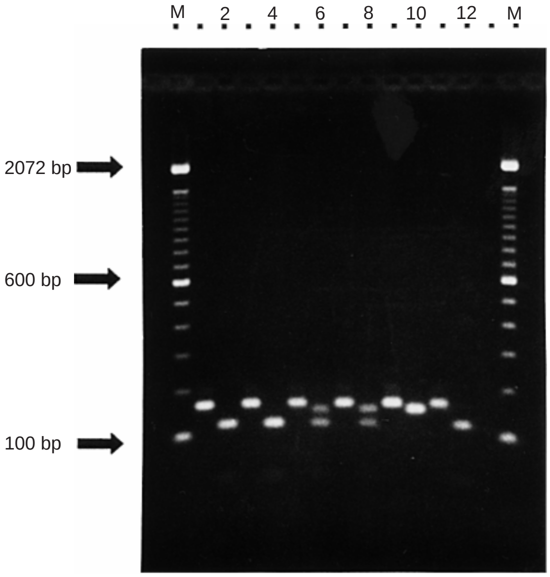

**Figure 2** Chromosome 12: RFLP of K-*ras*, codon 12 of exon 1 (patient 2). Legend: 100 bp ladder (M), normal mucosa (lane 1: PCR product; lane 2: PCR product digested with *Bst*NI), ACF (lane 3: PCR product; lane 4: PCR product digested with *Bst*NI), adenoma (lane 5: PCR product; lane 6: PCR product digested with *Bst*NI), carcinoma (lane 7: PCR product; lane 8: PCR product digested with *Bst*NI), $CaCo_2$ (lane 9: PCR product; lane 10: PCR product digested with *Bst*NI), CCL 228 (lane 11: PCR product; lane 12: PCR product digested with *Bst*NI), negative control (lane 13). The PCR product of 157 bp is digested twice in the case of wild-type K-*ras* yielding fragments of 114 bp, 29 bp and 14 bp (the small fragments are not visible on the gel). Mutation on codon 12 suppresses one restriction site thus yielding fragments of 143 bp and 14 bp after digestion with *Bst*NI. The tumour cell line $CaCo_2$ served as positive control (both alleles mutated), whereas CCL 228 has the wild-type sequence on codon 12

### Chromosome 2 (*hMSH2*)

Analyses of microsatellites of *hMSH2* showed deletions in the tissue samples of the malignant tumour in one allele of patient 1

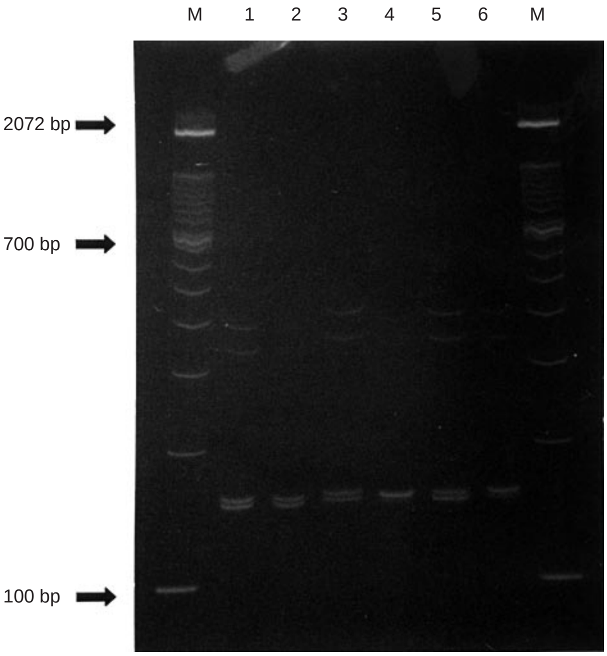

**Figure 3** Chromosome 17: pentanucleotide repeats at the p53 locus (patient 6). Legend: 100 bp ladder (M), normal mucosa (lane 1), ACF (lane 2), adenoma (lane 3), carcinoma (lane 4), normal liver (lane 5), liver metastasis (lane 6). Deletion of one allele is observed in the carcinoma (lane 4) and the liver metastasis (lane 6)

(locus D2S123) and patient 2 (D2S177, D2S134). In the protein truncation test, however, both patients showed physiologic hMSH2 proteins.

### *Chromosome 3 (*hMLH1*)*

Using the protein truncation test, aberrant proteins of *hMLH1* were not detected in these specimens.

### Chromosome 5 (*APC*)

Using heteroduplex analysis, we did not find any mutation in hot spot areas of the APC gene (exon 15, fragments E–G), but several deletions including the entire genomic region of the CA-repeats. Two deletions were detected in the adenoma of patient 2 (D5S82, D5S346), whereas the cancerous tissue did not reveal genomic alterations. In patient 3, the same deletions of microsatellites were found in adenomatous and cancerous tissue (D5S122, D5S346). Similarly, patient 4 presented two deletions of microsatellite regions in the adenocarcinoma, but only in one of the adenomas (D5S82, D5S346). The remaining three adenomas did not show loss of heterozygosity (LOH) on chromosome 5 (Figure 1). In patient 5, a deletion in the cancerous tissue was detected in one microsatellite locus (D5S82), whereas the adenoma appeared inconspicuous. Patient 6 presented no deletion at all.

### Chromosome 11 (H-*ras*)

None of the tissue samples showed mutations on codon 12 of the first exon of H-*ras*.

### Chromosome 12 (K-*ras*)

Point mutations of one allele were detected at codon 12 of the K-*ras* gene in patients 1 and 2. This mutation was observed in the

© 2000 Cancer Research Campaign

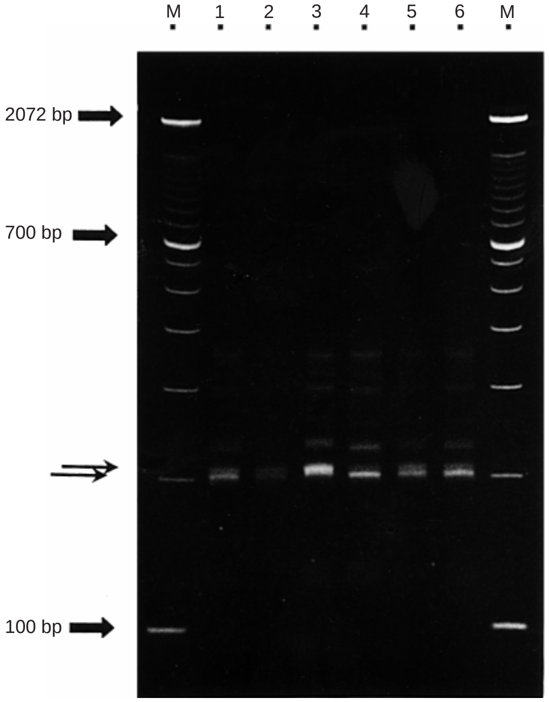

**Figure 4** Chromosome 18: variable number of tandem repeats at the DCC locus (patient 6). Legend: 100 bp ladder (M), normal mucosa (lane 1), ACF (lane 2), adenoma (lane 3), carcinoma (lane 4), normal liver (lane 5), liver metastasis (lane 6). The position of the alleles is marked by the double arrow showing the deletion of different alleles when comparing the adenoma and the carcinoma adenocarcinoma as well as in the adenoma of patient 2 (Figure 2). In patient 1, only the adenocarcinoma was affected. Codon 13 of K-*ras* was not mutated in these specimens.

### Chromosome 17 (*p53*)

Assaying SSCP as well as the formation of heteroduplices, we did not find mutations in hot spots of the *p53* gene (exons 5–8). In contrast to all four adenomas, a deletion within the penta-nucleotide motif (p53–5) was observed in patient 4. LOH of the dinucleotide (p53–2) was found in the adenocarcinoma of patient 3. In patient 6, both motifs (p53–2 and p53–5) were deleted in cancerous and metastatic tissue, whereas only the dinucleotide repeat p53–2 was deleted in the adenoma (Figure 3).

### Chromosome 18 (*DCC*)

No mutations were detected in codons 138–473 of the *DCC* gene by means of sequencing. Patients 1 and 2 manifested the wild-type gene of *DCC* and that of locus D18S34. Patients 3 and 4 presented one deletion in both the carcinoma and the adenoma. In patient 5, LOH of *DCC* in the adenocarcinoma was found. In patient 6 one allele of the *DCC* gene was deleted in the adenoma, whereas in the adenocarcinoma a corresponding deletion affected the other allele. It was noteworthy that the tissue of the metastasis showed the same pattern as observed in the carcinoma (Figure 4).

### Sequence of genetic alterations

The study of ACF did not identify lesions with genomic alterations. In patients 1 and 5, genetic defects were detected in the carcinoma only, but never in the precursor lesions. Accumulation of genetic aberrations was observed in patient 3, whose tumours presented allelic loss at *APC*- and *DCC*-associated loci in both the adenoma and the carcinoma. In addition, the adenocarcinoma showed two deleted regions of CA-repeats in proximity to the *p53* gene. The adenocarcinoma of patient 4 manifested five deletions, three of them also observed in the benign polyps. In contrast, independent tumorigenesis is suggested by the data of patients 2 and 6 by comparing the adenoma with the carcinoma. Although the co-existently appearing adenoma showed deletions within the *DCC* gene, different alleles were affected in patient 6. In patient 2, we found similar defects in the carcinoma and the adenoma on chromosome 12 (K-*ras*), but different deleted CA-repeats in the adenoma and carcinoma (Table 2).

### DISCUSSION

In our study, we investigated different synchronous lesions of the adenoma–carcinoma sequence of the colon. By applying SSCP, heteroduplex analysis, RFLP, sequencing and protein truncation tests, we studied a total of 25 loci relevant for mutational alter-

**Table 2** Genetic alterations observed in adenomas and carcinomas

| Patient | Adenocarcinoma | | | | | | Adenoma | | | | | |
|---|---|---|---|---|---|---|---|---|---|---|---|---|
| no. | *hMSH2*[a] | *hMLH1* | *APC* | *K-ras* | *p53* | *DCC* | *hMSH2* | *hMLH1* | *APC* | *K-ras* | *p53* | *DCC* |
| 1 | LOH | ND | ND | MUT | ND | ND | – | – | – | – | – | – |
| 2 | LOH | ND | ND | MUT | ND | ND | ND | ND | LOH | MUT | ND | ND |
| 3 | ND | ND | LOH | ND | LOH | LOH | ND | ND | LOH | ND | ND | LOH |
| 4 | ND | ND | LOH | ND | LOH | LOH | ND | ND | No. 1: ND | ND | ND | LOH |
| | | | | | | | | | No. 2: ND | ND | ND | ND |
| | | | | | | | | | No. 3: ND | ND | ND | ND |
| | | | | | | | | | No. 4: LOH | ND | ND | ND |
| 5 | ND | ND | LOH | ND | ND | LOH | ND | ND | No. 1: ND | ND | ND | ND |
| | | | | | | | | | No. 2: ND | ND | ND | ND |
| 6 | ND | ND | ND | ND | LOH | LOH allele a | ND | ND | ND | ND | LOH | LOH allele b |

ND = not detected, – = no material available, LOH = Loss of heterozygosity, MUT = Mutation of codon 12 on K -*ras*. [a]Although LOH was observed in proximity to the *hMSH2* locus, the repair protein was normal in the protein truncation test.

 © 2000 Cancer Research Campaign

**Table 3** Overview of genetic alterations observed in different types of tissue

| Patient no. | Normal mucosa | ACF | Adenoma | Carcinoma | Metastasis |
|---|---|---|---|---|---|
| 1 | None | None | – | *hMSH2*, K-*ras* | – |
| 2 | None | None | *APC*, K-*ras* | *hMSH2*, K-*ras* | – |
| 3 | None | None | *APC*, DCC | APC, p53, DCC | – |
| 4 | None | – | No. 1: DCC<br>No. 2: none<br>No. 3: none<br>No. 4: *APC* | APC, p53, DCC | – |
| 5 | None | None | No. 1: none<br>No. 2: none | *APC, DCC* | – |
| 6 | None | None | *p53, DCC*$_{\text{allele b}}$ | *p53, DCC*$_{\text{allele a}}$ | *p53*, *DCC*$_{\text{allele a}}$ |

– = no material available.

ations, potential deletions and microsatellite instabilities. We looked for these genetic alterations in ACF, adenomas differing in size and grade of dysplasia and adenocarcinomas with identical degree of differentiation. According to the adenoma-carcinoma sequence described by Fearon and Vogelstein (1990), we found an escalation of genetic aberrations in four of six patients when assaying synchronous lesions of the colon. All carcinomas were affected more frequently when compared with the corresponding adenomas. Although we investigated 25 different genetic markers, we were not able to unravel genetic differences between ACF and normal mucosa. This might indicate the presence of hidden mutations in not typical loci. The investigated ACF did not present any dysplasia. Recently, two pathways for the further development of ACF were described (Gregorio et al, 1997): one direction leads to dysplastic ACF, which are thought to develop in adenomas; the other direction describes the development to hyperplastic polyps. Thus, the absence of genetic alterations in our ACF is compatible with the latter hypothesis, which describes non-dysplastic ACF as precursors of hyperplastic polyps rather than of adenomas.

According to the adenoma–carcinoma sequence one would expect an increase of genetic alterations with size and decreasing differentiation of the lesions. Patient 4 with multiple colonic adenomas presented four larger adenomas differing in size and in degree of dysplasia. Only the smallest adenoma with mild dysplasia showed LOH in microsatellite regions of APC-associated loci (D5S82, D5S346), present also in the corresponding carcinoma of the rectum. In the largest and moderately differentiated adenoma, allelic deletion of the DCC-VNTR locus was detected. In contrast, the remaining intermediately sized adenomas were not genetically altered. In patient 6, the adenocarcinoma and the adenoma presented deletions of the *DCC* gene, but different alleles were affected. The liver metastasis showed the identical pattern as observed in the primary carcinoma.

In accordance with the suggested adenoma–carcinoma sequence of the colon, four patients reflected the progressive accumulation of genetic defects in synchronously appearing tumours during carcinogenesis. However, two patients with non-hereditary malignomas presented different genetic instabilities in different but synchronously appearing tumours, which indicates independent and simultaneous tumorigenesis within the same organ (Table 3). According to the linear model of colorectal carcinogenesis we assume that ACF and adenomas hide unknown genetic aberrations different from the investigated 25 loci. This study shows that although different tumours arise in an almost identical environment, their development is individual and linked to differential genetic alterations.

Disregarding hitherto unknown genetic mutations, we found in our study variations not compatible with linear models of carcinogenesis. Along this line, chaodynamical models were suggested recently to describe carcinogenesis by non-linear models (Schwab and Pienta, 1996; Sedivy, 1996*a*; Posadas et al, 1996; Coffey, 1998; Ferreira et al, 1998; Waliszewski et al, 1998). If this is the case, we propose non-linear models to explain the obtained heterogeneity observed in our results. Genetic instability among cells produces a tremendous and chaotic diversity that may lead to cancer (Coffey, 1998). From a holistic point of view, not only genetic events may drive the onset of cancer as suggested by Waliszewski (1998). In biology, deterministic and non-deterministic phenomena co-exist. Alterations of the dynamic cellular network and thermodynamic instability may contribute to the development of cancer (Waliszewski, 1997). The resulting unstable stationary state facilitates the chaotic dynamics leading to the fractal appearance of tumours (Cross, 1994; Sedivy, 1996*b*; Waliszewski, 1997). Chaotic diversity permits the tumour to produce a cell clone able to escape therapeutic influences (Coffey, 1998) as shown for prostate cancer (Posadas et al, 1996). Thus, understanding cancer as a complex adaptive system (Schwab and Pienta, 1996) and by means of chaos theory will help to establish new therapeutic concepts (Sedivy and Mader, 1997).

By finding low-level aberrations in the carcinoma of patient 2 (genetic instability on loci D2S177, D2S134; mutation of K-*ras* exon 12), which probably are not sufficient for malignant transformation, non-linear models of carcinogenesis might be applied. Although we investigated only a few patients, the observed exceptions urges additional explanations, if no hidden mutations exist.

In conclusion, in our study we found in synchronously appearing neoplastic lesion of the colon hints for the linear and clonal development of cancer. In contrast to this widely acclaimed model, we observed exceptions that either contain unknown genetic defects or might be explainable by a non-linear and holistic approach. Nevertheless, the small number of patients precludes detailed explanations. The trend of this data, however, urges to include alternative sights such as chaodynamical perspectives, which might help to understand these exceptions. Noteworthy, it is typical for chaodynamical processes that they appear at first sight as pure stochastic processes, but further investigations uncover their non-linear nature at least.

© 2000 Cancer Research Campaign

### REFERENCES

Boland CR, Sato J, Saito K, Carethers JM, Marra G, Laghi L and Chauhan DP (1998) Genetic instability and chromosomal aberrations in colorectal cancer: a review of the current models. *Cancer Detect Prev* **22**: 377–382

Breukel C, Tops C, van Leeuwen C, van der Klift H, Nakamura Y, Fodde R and Khan PM (1991*a*) CA repeat polymorphism at the D5S82 locus, proximal to adenomatous polyposis coli. *Nucleic Acids Res* **19**: 5804

Breukel C, Tops C, van Leeuwen C, van der Klift H, Nakamura Y, Fodde R and Khan PM (1991*b*) AT repeat polymorphism at the D5S122 locus tightly linked to adenomatous polyposis coli. *Nucleic Acids Res* **19**: 6665

Bufill JA (1990) Colorectal cancer: evidence for distinct genetic categories based on proximal or distal tumor location. *Ann Intern Med* **113**: 779–788

Cawkwell L, Lewis FA and Quirke P (1994) Frequency of allele loss of *DCC, p53, RB1, WT1, NF1, NM23* and *APC/MCC* in colorectal cancer by fluorescent multiplex polymerase chain reaction. *Br J Cancer* **70**: 813–818

Coffey DS (1998) Self-organization, complexity and chaos: the new biology for medicine. *Nat Med* **4**: 882–885

Cross SS, Bury JP, Silcocks PB, Stephenson TJ and Cotton DW (1994) Fractal geometric analysis of colorectal polyps. *J Pathol* **172**: 317–323

Fearon ER and Vogelstein B (1990) A genetic model for colorectal tumourigenesis. *Cell* **61**: 759–767

Ferreira SC, Martins ML and Vilela MJ (1998) A growth model for primary cancer. *Physica A* **261**: 569–580

Friedl W, Mandl M and Sengteller M (1993) Single-step screening method for the most common mutations in familial adenomatous polyposis. *Hum Mol Gen* **2**: 1481–1482

Gregorio CDI, Losi I, Fante R, Modica S, Ghidoni M, Pedroni M, Tamassia MG, Gafà L, Ponz de Leon M and Roncucci L (1997) Histology of aberrant crypt foci in the human colon. *Histopathology* **30**: 328–334

Iniesta P, deJuan C, Caldés T, Vega F-J, Massa M-J, Cerdán F-J, López J-A, Fernándex C, Sánchez A, Torres A-J, Balibrea J-L and Benito M (1998) Genetic abnormalities and microsatellite instability in colorectal cancer. *Cancer Detect Prev* **22**: 383–395

Jiang W, Kahn SM, Guillem JG, Lu S-H and Weinstein B (1989) Rapid detection of ras oncogenes in human tumours: application to colon, esophageal, and gastric cancer. *Oncogene* **4**: 923–928

Jones MH and Nakamura Y (1992) Detection of loss of heterozygosity at the human TP53 locus using a dinucleotide repeat polymorphism. *Genes Chromosomes Cancer* **5**: 89–90

Konishi M, Kikuchi-Yanoshita R, Tanaka K, Muraoka M, Onda A, Okumura Y, Kishi N, lwama T, Mori T, Koike M, Ushio K, Chiba M, Nomizu S, Konishi F, Utsunomiya J and Miyaki M (1996) *Gastroenterology* **111**: 307–317

Luce MC, Marra G, Chauhan DP, Laghi L, Carethers JM, Cherian SP, Hawn M, Binnie CG, Kam-Morgan LN, CayouetteMC, Koi M and Boland R (1995) In vitro transcription/translation assay for the screening of *hMLH1* and *hMSH2* mutations in familial colon cancer. *Gastroenterology* **109**: 1368–1374

Maesawa C, Tamura G, Suzuki Y, Ogasawara S, Sakata K, Kashiwaba M and Satodate R (1995) The sequential accumulation of genetic alterations characteristic of the colorectal adenoma–carcinoma sequence does not occur between gastric adenoma and adenocarcinoma. *J Pathol* **176**: 249–258

Miyake S, Nagai K, Yoshino K, Oto M, Endo M and Yuasa Y (1994) Point mutations and allelic deletion of tumour suppressor gene DCC in human esophageal squamous cell carcinomas and their relation to metastasis. *Cancer Res* **54**: 3001–3010

Posadas EM, Criley SR and Coffey DS (1996) Chaotic oscillations in cultured cells: rat prostate cancer. *Cancer Res* **56**: 3682–3688

Pretlow TP, Barrow BJ, Ashton WS, O'Riordan MA, Pretlow TG, Jurcisek JA and Stellato TA (1991) Aberrant crypts: putative preneoplastic foci in human colonic mucosa. *Cancer Res* **51**: 1564–1567

Schwab ED and Pienta KJ (1996) Cancer as a complex adaptive system. *Med Hypotheses* **47**: 235–241

Sedivy R (1996*a*) The potential role of apoptosis (programmed cell death) in a chaotic determined carcinogenesis. *Med Hypotheses* **46**: 455–457

Sedivy R (1996*b*) Fractal tumours: their real and virtual images. *Wien Klin Wochenschr* **108**: 547–51

Sedivy R and Mader RM (1997) Fractals, chaos, and cancer: do they coincide? *Cancer Invest* **15**: 601–607

Spirio L, Joslyn G, Nelson L, Leppert M and White R (1991) A CA repeat 30–70 kb downstream from the gene. *Nucleic Acids Res* **19**: 6348

Thibodeau SN, Bren G and Schaid D (1993) Microsatellite instability in cancer of the proximal colon. *Science* **260**: 816–819

Waliszewski P (1997) Complexity, dynamic cellular network, and tumorigenesis. *Pol J Pathol* **48**: 235–241

Waliszewski P, Molski M and Konarski J (1998) On the holistic approach in cellular and cancer biology: nonlinearity, complexity, and quasi-determinism of the dynamic cellular network. *J Surg Oncol* **68**: 70–78

Weber JL and May PE (1990) Dinucleotide repeat polymorphism at the D18S34 locus. *Nucleic Acids Res* **18**: 2201

Weissenbach J (1992*a*) *H. sapiens* (D2S123) DNA segment containing (CA) repeat. GenBank accession# Z16551

Weissenbach J (1992*b*) *H. sapiens* (D2S134) DNA segment containing (CA) repeat. GenBank accession# Z16763

Weissenbach J (1992*c*) *H. sapiens* (D2S177) DNA segment containing (CA) repeat. GenBank accession# Z17191

*© 2000 Cancer Research Campaign*
